# Deep brain stimulation in Early Onset Parkinson's disease

**DOI:** 10.3389/fneur.2022.1041449

**Published:** 2022-11-17

**Authors:** Patricia Krause, Johanna Reimer, Jonathan Kaplan, Friederike Borngräber, Gerd-Helge Schneider, Katharina Faust, Andrea A. Kühn

**Affiliations:** ^1^Movement Disorder and Neuromodulation Unit, Charité University Medicine Berlin, Berlin, Germany; ^2^Department of Neurosurgery, Charité University Medicine Berlin, Berlin, Germany

**Keywords:** early onset, Parkinson's disease (PD), deep brain stimulation (DBS), Parkinson's genes, quality of life

## Abstract

**Introduction:**

Subthalamic Deep Brain Stimulation (STN-DBS) is a safe and well-established therapy for the management of motor symptoms refractory to best medical treatment in patients with Parkinson's disease (PD). Early intervention is discussed especially for Early-onset PD (EOPD) patients that present with an age of onset ≤ 45–50 years and see themselves often confronted with high psychosocial demands.

**Methods:**

We retrospectively assessed the effect of STN-DBS at 12 months follow-up (12-MFU) in 46 EOPD-patients. Effects of stimulation were evaluated by comparison of disease-specific scores for motor and non-motor symptoms including impulsiveness, apathy, mood, quality of life (QoL), cognition before surgery and in the stimulation ON-state without medication. Further, change in levodopa equivalent dosage (LEDD) after surgery, DBS parameter, lead localization, adverse and serious adverse events as well as and possible additional clinical features were assessed.

**Results:**

PD-associated gene mutations were found in 15% of our EOPD-cohort. At 12-MFU, mean motor scores had improved by 52.4 ± 17.6% in the STIM-ON/MED-OFF state compared to the MED-OFF state at baseline (*p* = 0.00; *n* = 42). These improvements were accompanied by a significant 59% LEDD reduction (*p* < 0.001), a significant 6.6 ± 16.1 points reduction of impulsivity (*p* = 0.02; *n* = 35) and a significant 30 ± 50% improvement of QoL (*p* = 0.01). At 12-MFU, 9 patients still worked full- and 6 part-time. Additionally documented motor and/or neuropsychiatric features decreased from *n* = 41 at baseline to *n* = 14 at 12-MFU.

**Conclusion:**

The present study-results demonstrate that EOPD patients with and without known genetic background benefit from STN-DBS with significant improvement in motor as well as non-motor symptoms. In line with this, patients experienced a meaningful reduction of additional neuropsychiatric features. Physicians as well as patients have an utmost interest in possible predictors for the putative DBS outcome in a cohort with such a highly complex clinical profile. Longitudinal monitoring of DBS-EOPD-patients over long-term intervals with standardized comprehensive clinical assessment, accurate phenotypic characterization and documentation of clinical outcomes might help to gain insights into disease etiology, to contextualize genomic information and to identify predictors of optimal DBS candidates as well as those in danger of deterioration and/or non-response in the future.

## Introduction

Deep Brain Stimulation (DBS) of the subthalamic nucleus (STN) is a well-established treatment option for advanced Parkinson's Disease (PD) improving motor symptoms, quality of life and allowing to reduce dopaminergic medication (1). Aiming at a more symptom-specific, individualized DBS treatment there is a great interest to identify biomarkers that help to predict and monitor the individual DBS outcome. Moreover, personalized treatment decisions based on the individual status of the patient including genetics would be important not only for patients and their caregivers, but also for treating physicians when coucelling their patients. Studies like the EARLY STIM trial have proven that DBS in PD must not be limited to patients in advanced stages but rather ought to be offered to patients after the first years of motor fluctuations or dyskinesia with insufficient medical treatment adjustments (2). The significant beneficial impact of DBS on quality of life (QoL) in patients as well as the verification of long-term safety in different studies changed the clinical field toward an earlier approach in patients with motor fluctuations (2, 3). However, it is still difficult to predict patients' individual disease progression and potential functional DBS response. PD patients, *per se*, present heterogeneously in their clinical picture, their rate of disease progression and possible cognitive decline (4). While patients with dyskinesia and tremor, shorter disease duration, younger age, absence of dementia and stable psychiatric conditions seem to benefit particularly well from DBS, a predominance of axial symptoms and ON-Freezing correlates with presumably worse outcome (5–7).

Specific gene mutations seem to exert an individually different impact on disease progression, but also on DBS effects (8–10). PD patients with glucocerebrosidase (GBA) gene variants, e.g., present with faster progressing motor as well as cognitive deterioration (11, 12). Accordingly, although GBA patients benefit from DBS with regard to motor impairment and QoL (13, 14) they also experience faster worsening of cognitive decline and non-motor symptoms after DBS when compared to e.g., mutation-negative DBS patients (13–15). Parkin gene (PRKN) mutation has clinically been associated with a higher frequency and severity of impulse control behaviors (ICB) (16), but PRKN carriers seem to be good responders to DBS needing intervention later than GBA-gene mutation carriers (8). A more variable picture has been reported for LRRK2 mutation carriers where LRRK2-G2019S variant carriers show excellent STN-DBS responses (8, 17, 18), but stimulation effects in patients with LRRRK2-R1441G mutations was poor (8, 17).

At least 5–10% of the so-called Early-onset PD patients (EOPD) are estimated to carry genetic mutations (19, 20). With slight variability in literature, EOPD present first PD motor symptoms between the 21^st^ and 40–55^th^ year of age (21–23). Due to the comparably young age of onset (AOO) in EOPD, patients are more likely to still work for a living and take care of children. Several studies report on a correlation between younger age of onset and poor QoL in EOPD due to, e.g., difficulties with family and spouse, role expectations, a higher level of stigma, social isolation and job loss next to mere physical limitations (24–27). EOPD with DBS exhibit a higher percentage of PD gene mutations than their older counterparts (13, 28, 29). Besides, EOPD with proven gene mutations often show additional clinical features that differ from sporadic late onset PD (19, 30). Genetic factors, e.g., have been suggested to play a role in the development of early and/or severe dyskinesia with marked levodopa sensitivity as well as dystonia, painful cramps and dysautonomia (9, 10, 31–35). Furthermore, psychiatric symptoms such as depression, impulsive- or obsessive-compulsive behavior, increased intake of dopamine agonists (DA) or levodopa as well as substance abuse or dependence and paranoid symptoms is more common in EOPD (26, 35–37). Given the variable clinical presentation and levodopa response in EOPD with and without gene mutation (Mut+ and Mut-), treatment response to DBS might differ due to gene-specific effects. How much and in what way genetic status impacts the outcome of STN-DBS in PD is yet to be elucidated. Some studies indicate more durable results in patients with EOPD and certain PD gene mutations (38). A more recent review on DBS effects in monogenic PD patients, however, reported on rather variable cognitive and neuropsychiatric DBS benefits depending on the respective mutated gene (8) but no difference in DBS motor effects in monogenetic PD patients in comparison to the general PD population (8, 39). Especially beyond the most common LRRK2-, PRKN- and GBA-gene mutation, detailed knowledge and literature on the influence of individual monogenetic mutations on motor as well as non-motor DBS effects is limited. EOPD present with a highly complex clinical as well as psychosocial profile of requirement. Comprehensive data on DBS effects in this PD sub-cohort will be needed increasingly in order to offer individualized counseling and to weigh possible clinical benefits and risks of a highly elective surgical intervention in young patients with multi-layered treatment expectations.

Here, we retrospectively analyse DBS effects in a mere EOPD-cohort with 15% of proven PD-gene mutation carrier by means of standardized comprehensive clinical assessment and documentation of clinical outcome, socioeconomic aspects, non-motor symptoms as well as QoL data.

## Methods

### Patients and surgery

Patient selection was carried out by systematic search in the neuromodulation department's DBS database. Patients with the diagnosis of Parkinson's disease, with or without PD gene mutation, first clinical symptoms under the age of 45 years and deep brain stimulation were considered eligible to our study. Sixty-nine patients with EOPD and DBS met the inclusion criteria. Among those 23 patients that have been excluded from the present study, 8 patients did not have their 12 months follow-up at the time of data analysis, 6 patients had been lost to follow-up because of planned postoperative treatment in another center or relocation and 9 patients that had already been published in the EarlyStim-Study had been assessed with non-motor scales that were different to our protocol.

Retrospective analysis comprised pre- as well as postoperative follow-up data of 46 EOPD (27 men) operated in the subthalamic nucleus (STN-DBS) between 2012 and 2020. Patients had a mean disease duration of 12.9 ± 5.7 years and a mean age at surgery of 51.0 ± 8.8 years. Diagnosis was assured preoperatively corresponding to the British Brain Bank Criteria (40). PD-genes that were tested for comprised DNAJC13, DNAJC6, GBA, LRRK2, PANK2, PARK7, PINK1, POLG, PRKN, and SNCA. Since 2019, patients received even more comprehensive testing within the scope of the Rostock International Parkinson's Disease Study (ROPAD, www.clinicaltrials.com: NCT03866603). Among the present cohort, 7 patients presented with previously assured PD-gene mutation including 1 LRRK2-, 2 PARK2-, 1 PINK1-, 1 DJ1-, and 2 GBA-gene mutation carriers. Fourteen patients had undergone negative genetic testing for PD-associated mutations and 25 patients did not want to be tested or had not undergone genetic testing, respectively.

All patients received bilateral STN-DBS (7 patients: Model 3389; Medtronic, Minneapolis, MN; 36 patients: Model DB-2202; Boston Scientific Vercise Directional; Boston Scientific, Marlborough, MA; 3 patients: Modell DB-2201; Boston Scientific Vercise Non-Directional; Boston Scientific, Marlborough, MA) following the standard surgical procedure at our center, which is described in detail elsewhere (41). In short, multimodal high-resolution pre-operative magnetic resonance imaging (MRI) was performed in all patients to exclude anatomical comorbidities and for stereotactic planning. A Leksell Frame (Elekta, Stockholm, Sweden) was used for stereotactic targeting. A microelectrode drive with up to five test electrodes (Ben-gun array) was mounted to the frame to control the insertion of microelectrodes *via* an external device (Alpha Omega, Nazareth, Israel), while simultaneously recording multi-unit activity to determine STN/SnR borders. Intraoperative test stimulations were then performed to assess the therapeutic window on each test electrode. Findings were discussed by the interdisciplinary team before the permanent electrodes were positioned. CT scans were performed the following day to confirm correct electrode placement and to exclude postoperative hemorrhage. In the second stage (2–6 days after electrode implantation) the internal pulse generator (IPG) was implanted and connected to the leads (30 patients: Model Vercise PC and 9 patients: Model Vercise Gevia; Boston Scientific, Marlborough, MA; 2 patients: Model Percept PC and 5 patients: Modell Activa PC 37601; Medtronic, Minneapolis, MN). Standard stimulation parameters for STN-DBS directly after surgery include 130 Hz, 60 μs and 0.5–1 mA on the second lowest contacts. After subsiding stun effect at 3 months follow-up, careful selection of optimal contact and individual amplitude with highest threshold for side-effects and widest therapeutic window for beneficial stimulation effects is chosen in a thorough monopolar review.

### Clinical assessments

Preoperative motor assessment (baseline, BL) included performance of a standardized levodopa challenge by use of the UPDRS III without dopaminergic substitution for ≥12 h (Med-OFF) and after 200 mg of soluble levodopa (Med-ON) as well as assessment of the UPDRS II and IV (42). Non-motor evaluation encompassed the UPDRS I questionnaire (42) as well as the assessment of mood (BDI-II) (43), impulsiveness (QUIP) (44), apathy (Starkstein-Apathy-Score) (45) and quality of life (PDQ39) (46). Additional information was gathered on medical history with focus on dopaminergic therapy, cognition by means of MMSE (47) and DemTect (48), clinical history including family history and genetic background as well as extensive exploration of additional motor and neuropsychiatric features. Additional features investigated included presence of severe dystonia, gait disorders or dyskinesia as well as psychiatric symptoms such as depression, paranoia, hallucinations, levodopa-dysregulation, impulsive behavior and cognitive impairment. Levodopa equivalent daily dose (LEDD) conversion was calculated corresponding to Schade et al. (49). At 12-MFU the complete preoperative assessments were repeated. UPDRS III was explored under four different conditions with and without medication and stimulation, e.g. STIM-ON/MED-ON; STIM-OFF/MED-ON; STIM-ON/MED-OFF and STIM-OFF/MED-OFF, respectively. Additionally, postoperative adverse and severe adverse effects (AE and SAE), stimulation parameters after 3 and 12 months and information on impulse-generator (IPG) were gathered. All motor evaluations were video-documented. In cases of lacking sub-scores, the total patients' number (n) is given separately. The study was approved by the local Ethics Committee **(EA4/263/21)**. All patients gave their written informed consent.

### Statistical analysis

Pre- and postoperative scores were compared between BL and 12-MFU using the Wilcoxon test corrected for multiple comparisons. A Spearman's correlation was done in order to investigate possible correlations between motor outcome (UPDRS III STIM-ON/MED-OFF and STIM-OFF/MED-OFF at 12MFU) and age at onset, disease severity, non-motor symptoms (changes of mood, apathy, impulsivity, QoL) and initial levodopa-response as well as possible correlations between DA reduction and motor and non-motor improvements and in-between non-motor symptom changes (mood, apathy, impulsivity and QoL). Additionally, a sub-analysis comparing patients with and without PD gene mutation (*n* = 14 mut- and *n* = 7 mut+) was performed using the Wilcoxon Mann-Whitney test. In case of any missing values for comparison of pre- and postoperative scores, the total number of available and analyzed scores is given in numbers (*n*) explicitely. Since the study was of retrospective character, all analysis were performed with exploratory instead of confirmatory intent. All data are given as mean ± SD if not mentioned otherwise. A *P* value < 0.05 was considered to be significant.

## Results

### Clinical motor assessment

Mean motor scores (UPDRS III) had improved significantly after STN-DBS with 52.8 ± 23.4 points in MED-OFF condition at BL compared to 26.7 ± 16.2 points in STIM-ON/MED-OFF condition at 12 MFU (*p* < 0.001; *n* = 42). UPDRS III motor scores in MED-OFF condition at BL and STIM-OFF/MED-OFF-condition at 12-MFU remained stable with 52.8 ± 23.4 points vs. 52.4 ± 17.6 points (*p* = 0.98), respectively. STIM-OFF/MED-OFF vs. STIM-ON/MED-OFF at 12MFU improved from 52.4 ± 17.6 points to 26.7 ± 16.2 points (*p* < 0.001; *n* = 42). UPDRS IV improved significantly after 12 months by 4.9 ± 6.4 points (*p* = 0.0003; *n* = 29). The LEDD was significantly reduced by 58.5 ± 32.5% at 12-MFU compared to BL (see Table 1).

**Table 1:** Baseline (BL) as well as 12-months follow-up (12-MFU) results of motor and non-motor symptoms including mood, apathy, impulsiveness and cognition as well as PDQ-39-summary index representing quality of life changes in the total cohort of 46 Early-Onset-Parkinson's disease patients given in mean±standard deviation.

**Score**	**BL**	**12-MFU**	***p*-value**
UPDRS I	11.74 ± 7.5 pts.	9.1 ± 6.1 pts.	0.05*
UPDRS II	12.9 ± 8.3 pts.	11.8 ± 8.2 pts.	0.53
UPDRS III	52.8 ± 23.4 pts.	26.7 ± 16.2 pts.	< 0.001*
UPDRS IV	7.6 ± 5.6 pts.	2.7 ± 3.7 pts.	< 0.001*
LEDD	1,313.1 ± 598.5 mg/d	465.9 ± 354.7 mg/d	< 0.001*
BDI-II	13.6 ± 8.3 pts.	11.7 ± 7.5 pts.	0.28
Apathy	14.2 ± 5.1 pts.	14.6 ± 5.1 pts.	0.43
QUIP	13.4 ± 15.0 pts.	7.2 ± 9.2 pts.	0.017*
MMST	28.7 ± 1.7 pts.	28.5 ± 2.7 pts.	0.47
DemTect	14.2 ± 3.2 pts.	14.4 ± 4.2 pts.	0.53
PDQ-39	39 ± 15 pts.	28.8 ± 18 pts.	0.011*

UPDRS, United Parkinson's Disease Rating Scale; LEDD, daily levodopa dosage; BDI-II, Beck Depression Inventory; QUIP, Questionnaire for Impulsive-Compulsive Disorders in Parkinson's Disease; MMSE, Mini Mental State Examination; DemTect, Dementia Detection Test; PDQ39, 39-Item Parkinson's Disease Questionnaire; ^*^highlighting significant differences (p ≤ 0.05) between BL and 12-MFU scores.

### Changes in non-motor symptoms

**Figure 1:**
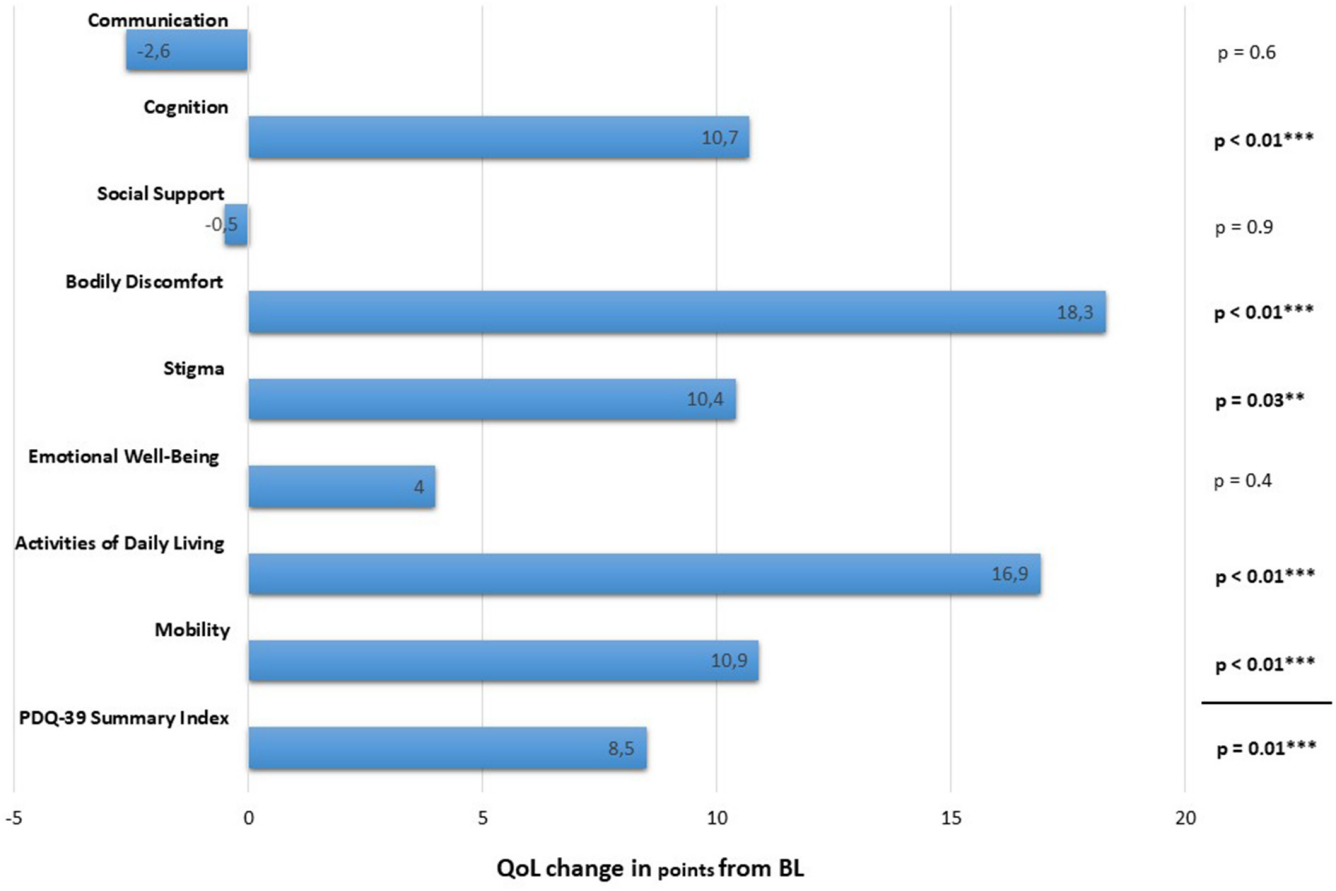
Quality of life as assessed by means of the Parkinson's Disease Questionnaire (PDQ-39) comparing absolute changes from baseline (BL) to 12 months follow-up (12-MFU) in all Early-Onset Parkinson's Disease patients with available pre- and postoperative scores (*n* = 33). Each column depicts the absolute change of subscores for the various domains of the PDQ-39 after 12 months of DBS. The first column indicates the overall absolute change in the summary index of the PDQ-39 at 12-MFU. Positive values indicate improvement. *highlights statistically significant changes *p ≤ 0.05; **p ≤ 0.03; ***p ≤ 0.01.

UPDRS I improved significantly after 12 months by 2.5 ± 7.5 (*p* = 0.045; *n* = 35). Changes in activities of daily living (UPDRS II) after 12 months of STN-DBS did not reach statistical significance (see Table 1). Neuropsychiatric evaluation for impulsive control disorder quantified by means of QUIP-Rating scale showed a significant reduction of 6.6 ± 16.1 points (*p* = 0.02; *n* = 35) after STN-DBS. PDQ39, with lower scores reflecting better QoL, improved significantly from 38.9 ± 14.9 points at BL to 28.8 ± 18.0 points at 12-MFU (*p* = 0.01; *n* = 33). Sub-analysis revealed statistically significant improvements covering the dimensions mobility, activities of daily living, stigma, cognition and bodily discomfort. No significant change was found for the dimensions emotional well-being, social support and communication (see Figure 1). Mood measured by means of the BDI-II- and apathy measured by the Starkstein-Apathy-Scale revealed no significant changes at 12-FU (13.6 ± 8.3 and 14.2 ± 5.7 points) in comparison to BL (11.7 ± 7.5 and 14.6 ± 5.1 points; *p* = 0.3 and *p* = 0.4), respectively. At baseline, altogether 64 motor and/or neuropsychiatric additional comorbidities were documented. After 12 months of DBS, these complaints had diminished to 17 (see **Table 3**). Cognition evaluated at BL and 12-MFU remained stable with 28.7 ± 1.7 vs. 28.5 ± 2.7 points in the MMSE (*p* = 0.47) and 14.2 ± 3.2 vs. 14.4 ± 4.2 points in the DemTect (*p* = 0.53).

### Correlation of motor and non-motor effects

Motor improvements 12 months after DBS correlated significantly with initial levodopa response in the initial BL-levodopa challenge (*p* = 0.001) and improvements of mood (*p* = 0.03) but not with apathy, impulsivity, cognition, QoL, DD or AOO. BDI changes correlated also with reduction of apathy, impulsivity and QoL at 12 MFU (*p* = 0.01, *p* < 0.01, and *p* < 0.01, respectively). Postoperative LEDD and DA reduction did neither correlate with motor nor with non-motor score changes.

### Subgroup analysis Mut+ (*n* = 7) vs. Mut- (*n* = 14) EOPD patients

With an AOO of 32.1 ± 10.7 yrs., an age of 41.3 ± 12 years at surgery and a disease duration of 9.3 ± 5.1 years, patients with PD-gene-mutation tended to be younger at surgery and to have a shorter disease duration (DD) in comparison to the Mut- patients (AOO 37.5 ± 7.1 yrs.; AAS 50.8 ± 9.5 years; DD 14 ± 6.3 yrs; *p* = 0.24, *p* = 0.11 and 0.1, respectively). None of these demographic differences reached statistical significance. At 12MFU, the Mut+ patients presented a significantly reduced LEDD (952.9 ± 611.3 mg/d vs. 469 ± 450.7 mg/d; *p* = 0.025) and motor score (UPDRS III: 51 ± 18 pts. vs. 32.1 ± 1.6 pts.; *p* = 0.025). Changes of non-motor symptoms in the Mut+ cohort did not change significantly (see Table 2). Despite significant measurable motor improvements the two PARK2+ patients experienced worsening of QoL at last follow-up mainly because of still intermittently occurring severe OFF dystonia and worsening in the QoL subdivisions “activities of daily living,” “social support,” and “communication.” One of them (see Supplementary Figure 1; yellow line), moreover, experienced significant worsening of apathy after DBS and a 75% reduction of levodopa. In comparison, the Mut- cohort presented a significant LEDD-reduction (1,274.9 ± 553.2 mg/d vs. 437 ± 373.2 mg/d; *p* < 0.01), significant improvements of the motor symptoms (UPDRS III, IV: 60.3 ± 23.3 pts. vs. 28.2 ± 20.5 pts.; *p* < 0.01 and 5.8 ± 5.2 pts. vs. 1.6 ± 3.4 pts.; *p* = 0.05, respectively) as well as significant changes of QoL (33.1 ± 10.3 pts. vs. 21.5 ± 16 pts.; *p* < 0.03) at 12-MFU. Non-motor symptoms in the Mut- cohort did not change significantly. Apathy scores did neither improve not deteriorate significantly in the Mut+ or the Mut- cohort. Subgroup-comparison, however, depicted a significant antidromic change in-between the Mut+ and Mut-subcohort after DBS (for detailed values see Table 2). LEDD and UPDRS III reduction as well as stimulation parameter at 3- and 12-MFU did not differ significantly between the two cohorts. No (S) AE was noted in the Mut+ cohort.

**Table 2:** Subcohort analysis of Early-Onset-Parkinson's patients with (Mut+) and without (Mut-) PD-gene mutation with regard to demographic differences, e.g. age of onset, disease duration and age at surgery, as well as baseline (BL) as well as 12-months follow-up (12-MFU) results of motor and non-motor symptoms including mood, apathy, impulsiveness and cognition and PDQ-39-summary index representing quality of life changes given in mean ± standard deviation.

	**EOPD-Mut** + **(n** = **7)**		**EOPD-Mut- (n** =**14)**		**p value (Mut+ vs. Mut-)**
Age of Onset	32.1 ± 10.7 years		37.5 ± 7.1 years		= 0.2
Disease Duration	9.3 ± 5.1 years		14 ± 6.3 years		= 0.1
Age at Surgery	41.3 ± 12 years		50.8 ± 9.6 years		= 0.1
LEDD at BL	952.9 ± 611.3mg/day		1274.9 ± 553.2mg/day		= 0.9
LEDD at 12MFU	469.6 ± 450.7mg/day**		437.0 ± 373.2mg/day***		
UPDRS I at BL	17.4 ± 3.9 points	n = 5	11.7 ± 5.8 points	n=12	= 0.5
UPDRS I at 12MFU	14.8 ± 7.5 points		7.1 ± 6.4 points**		
UPDRS II at BL	13.4 ± 10.1 points	n=5	10.5 ± 9.5 points	n=12	= 0.7
UPDRS II at 12MFU	16 ± 11 points		12.2 ± 8.7 points		
UPDRS III at BL	51.4 ± 18.0 points	n=7	60.3 ± 23.3 points	n=14	= 0.2
UPDRS III at 12MFU	32.1 ± 14.6 points**		28.2 ± 20.5 points***		
UPDRS IV at BL	3 ± 6 points	n=4	5.8 ± 5.2 points	n=9	= 0.1
UPDRS IV at 12MFU	2.2 ± 3.1 points		1.6 ± 3.4 points*		
BDI-II at BL	12.8 ± 9.7 points	n=7	12.1 ± 8.6 points	n=14	= 0.2
BDI-II at 12MFU	12.4 ± 7.7 points		9.6 ± 7.4 points		
Apathy at BL	12.3 ± 4.4 points	n=7	15.4 ± 7.5 points	n=13	= 0.03*
Apathy at 12MFU	16.3 ± 5.4 points		13.2 ± 6.0 points		
QUIP at BL	25.8 ± 25.6 points	n=6	6.5 ± 9.5 points	n=13	= 0.7
QUIP at 12MFU	11.6 ± 12.5 points		6.2 ± 10.2 points		
PDQ-39 Summary Score at BL	52.3 ± 25.1 points	n=5	33.1 ± 10.3 points	n=11	= 0.2
PDQ-39 Summary Score at 12MFU	43.8 ± 27.5 points		21.5 ± 16.0 points**		
MMST at BL	27.7 ± 1.4 points	n=6	29.57 ± 0.7 points	n=13	= 1.0
MMST at 12MFU	27.3 ± 3.3 points		28.2 ± 3.8 points		
DemTect at BL	14 ± 6.7 points	n=4	15.1 ± 2.6 points	n=9	= 0.7
DemTect at 12MFU	12.7 ± 5.7 points		14.8 ± 3.2 points		

n, number of patients; UPDRS, United Parkinson's Disease Rating Scale; LEDD, daily levodopa dosage; BDI-II, Beck Depression Inventory; QUIP, Questionnaire for Impulsive-compulsive Disorders in Parkinson's Disease; MMSE, Mini Mental State Examination; DemTect, Dementia Detection Test; PDQ39, 39-Item Parkinson's Disease Questionnaire; significant changes highlighted with asterisk, e.g., ^*^p = 0.05; ^**^p < 0.03; ^***^p < 0.01.

### Occupation

Preoperatively, 11 patients worked full- and 4 patients part-time. Thirty-one patients did not work at baseline with 25 patients being retired. Twelve months after STN-DBS, 9 patients still worked full- and 6 part-time. Among the remaining 31 patients, 28 patients were retired early due to PD and 3 not working for other reasons. QoL at baseline was 42.6 ± 15.2 pts. in those patients without employment before DBS and 31.5 ± 11.5 pts in those still occupied pre DBS (*p* = 0.02).

### Stimulation data and safety

All patients had received subthalamic deep brain stimulation. Three months after surgery mean stimulation settings were 2.4 ± 1.0 mA, 128.4 ± 9.5 Hz, 59.3 ± 3.3 μs for the left STN and 2.2 ± 0.9 mA, 154.7 ± 176.4 Hz, 59.8 ± 3.3 μs for the right STN. At 12-MFU the settings were 3.0 ± 1.0 mA, 133.6 ± 23.1 Hz, 57.6 ± 8 μs for the left STN and 2.8 ± 0.9 mA, 133.6 ± 23.1 Hz, 60 ± 9 μs for the right STN. In the first year after implantation, seven adverse events (AE) occurred including dyskinesia (3 patients), dysarthria (3 patients) and accidental STIM-OFF (1 patient). One patient with dyskinesia needed a transient stimulation pause directly after stimulation. Dyskinesia and dysarthria was each responsive to stimulation adjustments. In the patient with accidental STIM-OFF, no defect could be found after reactivation of stimulation and AE did not occur again. Nine serious adverse events (SAE) occurred in this cohort: One patient with impaired wound-healing improved under antibiotics, one patient needed 24 h observation time after a fall with associated head trauma but without damage to the system, three patients experienced postoperative confusion that resolved spontaneously after 3–5 days and one patient suffered from intracranial bleeding around one electrode with associated aphasia that remitted incompletely up to 12 MFU. One patient attempted suicide after newly started DA-therapy and resulting impulsivity 11 months after electrode implantation.

## Discussion

The present results demonstrate that EOPD with and without known genetic background benefit from STN-DBS with about 53% motor improvement measured by UPDRS III accompanied by a ~59% LEDD-reduction 12 months after implantation. In line with this, patients experienced a meaningful reduction of additional motor as well as non-motor neuropsychiatric symptoms corroborated by significant improvements in the UPDRS IV with regard to motor fluctuations and a significant reduction in the QUIP-Score examining impulsivity in PD. Furthermore, patients experienced significant 30% improvements of QoL measured by the PDQ-39.

Decision making on the right timing of an elective surgical procedure such as DBS ought to be done in a highly individualized manner and under careful weighting of motor complications, motor subtype, putative disease progression, gender, genetics, presence of specific symptoms at diagnosis, lifestyle and treatment preferences (50). In their review, Kleiner-Fisman et al. (51) described greater improvements after subthalamic DBS in PD-patients with higher baseline motor deficits, longer disease duration and a higher baseline levodopa responsiveness prior to surgery (52). Correspondingly, responsiveness to DBS in our EOPD-cohort correlated significantly with presurgical responsiveness of motor symptoms to levodopa. Additionally, our EOPD cohort has an overall high motor improvement compared to recent STN-DBS trials (53) and motor as well as non-motor benefits of our cohort are at least comparable if not better than those in advanced later onset PD-patients with regard to previous trials (51, 53–55). Accordingly, several studies describe a profound and persistent improvement of disability and motor features as well as relatively better axial and cognitive outcomes of operated patients with younger age (6, 56–59) which, to some extend, might derive from more efficient compensatory mechanisms in younger patients (60). Hitherto publication landscape on DBS in genetic and early onset PD puts a main emphasis on motor outcome. Although motor improvements self-evidently are important in PD-patients and we also found a correlation between motor improvements and changes in BDI in our cohort (*p* = 0.03), mood and hypo- as well as hyperdopaminergic behavior exert greater effects on QoL in PD than do motor complications (61). With regard to impulsivity, we realized a statistically significant reduction of impulsivity in the QUIP-score together with a meaningfully reduced declaration of additional neuropsychiatric symptoms under subthalamic stimulation and after significant overall LEDD-reduction at 12 MFU (see Tables 1, 3). This is in line with data on STN-DBS-induced alleviation of neuropsychiatric non-motor fluctuations and hyperdopaminergic behaviors that could also be seen in comparison to best medical PD treatment in the EARLYSTIM cohort (62, 63). ICB is observed under higher dosages of pulsatile L-Dopa monotherapy (64). In allowing the reduction of dopaminergics, STN-DBS can help to reduce not only dyskinesia that is frequent in EOPD but also to improve psychiatric side effects based on dopaminergic overtreatment (57, 62, 63, 65). Hassan et al. found that men were more likely to develop DA-induced ICBs (66). Due to a higher D3-receptor-affinity, especially DA dose reduction is reported to significantly diminish hyperdopaminergic behaviors (62, 67). Mere DA component reduction of the total LEDD alone did not explain the beneficial neuropsychiatric benefits, in our cohort. However, one patient of our cohort without known gene mutation and a tendency for ICB became hypomanic after re-initiation of DA-therapy 9 months after STN-DBS and attempted suicide 8 weeks later in association to a marital dispute. DA-withdrawal resolved ICB and suicidal tendencies in this patient so that DBS was not considered responsible for the suicide attempt. Another of our patients had attempted suicide before DBS due to DA-associated hypersexuality and severe motor fluctuations. After STN-DBS with significantly reduced LEDD and relevant motor benefits this patient never complained of suicidal ideation again to date. Additionally, the PDQ39-subitem “cognition” had improved significantly after LEDD reduction in our EOPD cohort at 12 MFU. While neuropsychiatric off-drug fluctuations are associated with e.g. reduced motivation, empty mind and cognitive slowing, on-drug effects might induce racing of thought (67). Studies comparing EOPD and older-onset PD-patients found that PD-patients with younger AOO show worse QoL as well as depression scores and perceive more stigmatization than older patients with comparable disease severity or disability (27). With regard to this, not only “cognition” but also the QoL subdivisions “mobility,” “activities of daily living,” “stigma,” and “bodily discomfort,” each representing a potential prerequisite of participation in social and professional life, improved significantly under STN-DBS in our cohort. Moreover, improvements of mood correlated significantly with lower apathy and impulsivity scores (*p* = 0.01 and *p* < 0.01, respectively) and higher QoL (*p* < 0.01).

**Table 3:** Additional motor and/or neuropsychiatric patient complaints and symptoms apart from bradykinetic-rigid syndrome or tremor in the EOPD cohort at baseline (BL) and after 12 months of DBS (12-MFU).

	**BL (*n*)**	**12-MFU (*n*)**
OFF-dystonia	13	6
Dyskinesia	12	2
Gait difficulties	2	1
Impulse control disorder	16	2
Depression	8	6
Paranoia	5	0
Subjective cognitive slowing/ reduced cognitive impairment	3	0
Hallucinations	3	0
Levodopa disinhibition	2	0

61% of our cohort were already retired aged 51 ± 9 years at baseline and showed significantly worse initial QoL scores than those still working. Having said that, the initial 32% of fully employed patients were still working 12 months after DBS, so that one might speculate on potential benefits of earlier DBS on coping strategies in question of professional abilities. Additionally, QoL subdivisions “social support,” “communication,” and “emotional well-being” possibly affecting working abilities as well, have not been improved significantly after 12 months of DBS in our cohort. EOPD are confronted with a variety of additional challenges such as disruption of family life, depression, social isolation, greater perceived stigmatization and disability-related loss of income (27, 68). Preliminary studies on employment in EOPD found a mean time to loss of employment of 4.9 years. 46% of young PD-patients had stopped working after a disease duration of 5 years and 82% after 10 years of PD (23). Reversal of retirement is not to be expected so that early medical as well as social support and possibly earlier intervention might help to improve the rate of full occupation in EOPD patients.

So far, knowledge on the genetic background of PD-patients leads to no specific recommendations in the clinical evaluation procedure for DBS. In our cohort, 7 patients presented with known PD-gene mutations. In direct comparison to the Mut- patients, our Mut+ cohort tended to have a shorter disease duration at time of surgery. Angelini et al. found significantly differing disease durations between genetic subgroups in PD-DBS patients (13). While in our subanalysis comparison of Mut+ and Mut- EOPD revealed no significant difference in motor outcome, mean UPDRS III motor improvement in the Mut+ EOPD reached only ~35% most likely due to the more variable motor response in this small patient cohort. Additionally, the two PARK2+ patients deteriorated with regard QoL at last follow-up due to residual OFF-dystonia, development of severe apathy in one patient (see suppl. fig. 1; yellow line) and worsening of the PDQ39 subdivisions “activities of daily living,” “social support,” and “communication.” One additional complicating factor in this context might have been the immigrant status with severe language barrier in both patients.

Several studies found that patients with PD-associated mutations benefit from STN-DBS as well as patients without known PD gene-mutation (69, 70). Moro et al. found a reduced DBS effect in the mutation carrier group after 12 months of stimulation. However, this difference vanished after 3–6 years of continuous stimulation due to a more pronounced clinical decline of the non-mutation carriers after 3-5 years (71). Apathy changed significantly in-between the Mut+ and Mut-cohort in our study but worsened in the Mut+ and improved in the Mut- patients after DBS (see Table 2) with a tendency to worsening in the Mut+. A correlation with DA-reduction as an explanation for this in-between subgroup-trend could not be found. For the two patients with less favorable outcome (see Supplementary Figure 1) case 6 had a GBA mutation and case 7 was identified as a LRRK2-(p.G2019S-variant) mutation carrier. The latter variant has been associated with favorable outcome in previous study (8). Kuusimäki et al. (8) summarize previous study results reporting on poor DBS-outcome in LRRK2-patients with p.R1441G mutations compared to excellent results in p.G2019S-mutation carriers, development of neuropsychiatric problems 5–7 years after DBS in two p.T2031S-mutation carriers, inhomogeneous clinical benefits with regard to GBA-gene mutation and development of cognitive and/or neuropsychiatric problems some years after implantation in 3/5 SNCA-mutation carriers. Thus, certain genetic isoforms might have an impact on DBS effect.

Adverse events associated with DBS are of crucial importance in consideration for early DBS that needs weighing up of possible clinical benefits against individualized risks, requirements of the surgical procedure with IPG changes, possible lead fractures or malfunctioning and the lifelong need for follow ups by an experienced multidisciplinary team: SAE frequency in our cohort with overall 6.5% (*n* = 3) patients with postoperative confusion, 2.2% with wound healing difficulties and 2.2% intracranial bleeding (*n* = 1, respectively) did not outnumber previously published DBS risks (72).

We here present a single center EOPD-cohort treated with the same standardized procedures by the same team. The main strength of this study lies in the detailed information on motor and non-motor symptom evaluation by means of the UPDRS I-IV, documentation of additional neurological as well as neuropsychiatric features and information on occupation, mood, cognition, QoL, genetic status, stimulation parameters and (S)AES in 46 patients with EOPD and STN-DBS. The panels used here are convenient for use in the routine outpatient clinic allowing standardization and easy implementation in the clinical setting. Although our cohort size is one of the largest in studies of this kind, it is still small in terms of absolute numbers. Further limitations of the study lie in the rather short follow-up period up to now, the isolated use of STN as a target, the retrospective design, the monocentric study, the small number of mutation carriers, the unequal distribution of Mut+ and Mut- patients as well as in the fact that not all patients underwent genetic testing for PD mutations. A better understanding of the genetic background and associated clinical features might have an impact on decision making in DBS and the eventual individual outcome. Proper patient characterization is becoming increasingly relevant. Longitudinal monitoring of DBS-EOPD-cohorts over long-term periods with standardized comprehensive clinical assessment, accurate phenotypic characterization and documentation of clinical outcome might help to gain insights into disease etiology, to contextualize genomic information and to identify predictors of optimal EOPD-DBS candidates as well as those in danger of deterioration and/or non-response in future. Therefor our cohort encouraged us to establish the so-called EOPS-DBS-Registry (DRKS00028134) in order to gain knowledge on patients' progression and long-term outcome that might enable clinicians to improve the counseling of EOPD-patients.

## Data availability statement

The original contributions presented in the study are included in the article/Supplementary material, further inquiries can be directed to the corresponding author.

## Ethics statement

The studies involving human participants were reviewed and approved by Local Ethics Committee, Charité [EA4/263/21]. All patients gave their written informed consent.

## Author contributions

AK, PK, JR, JK, FB, G-HS, and KF: conception and design of the study, or acquisition of data, or analysis and interpretation of data. PK, JR, JK, G-HS, KF, and AK: drafting the article or revising it critically for important intellectual content. PK and AK: final approval of the version to be submitted. All authors contributed to the article and approved the submitted version.

## Funding

This study was funded by the Deutsche Forschungsgemeinschaft (DFG, German Research Foundation) – Project-ID 424778381 – TRR 295. KF is supported by the BIH Clinical Fellow Programm, Charité.

## Conflict of interest

Author AK received speaker's honoraria and consultancies from Medtronic and Boston Scientific. Author PK received speaker's honoraria from Medtronic and is in the Advisory Board of Abbott, outside the submitted work. Author G-HS received speaker's honoraria from Medtronic, Boston Scientific and Abbott. The remaining authors declare that the research was conducted in the absence of any commercial or financial relationships that could be construed as a potential conflict of interest.

## Publisher's note

All claims expressed in this article are solely those of the authors and do not necessarily represent those of their affiliated organizations, or those of the publisher, the editors and the reviewers. Any product that may be evaluated in this article, or claim that may be made by its manufacturer, is not guaranteed or endorsed by the publisher.
