# Supplementary figures and images for: Deep brain stimulation in Early Onset Parkinson's disease
Source: Front Neurol. 2022 Nov 17;13:1041449. doi: 10.3389/fneur.2022.1041449 (PMC9713840; doi:10.3389/fneur.2022.1041449)

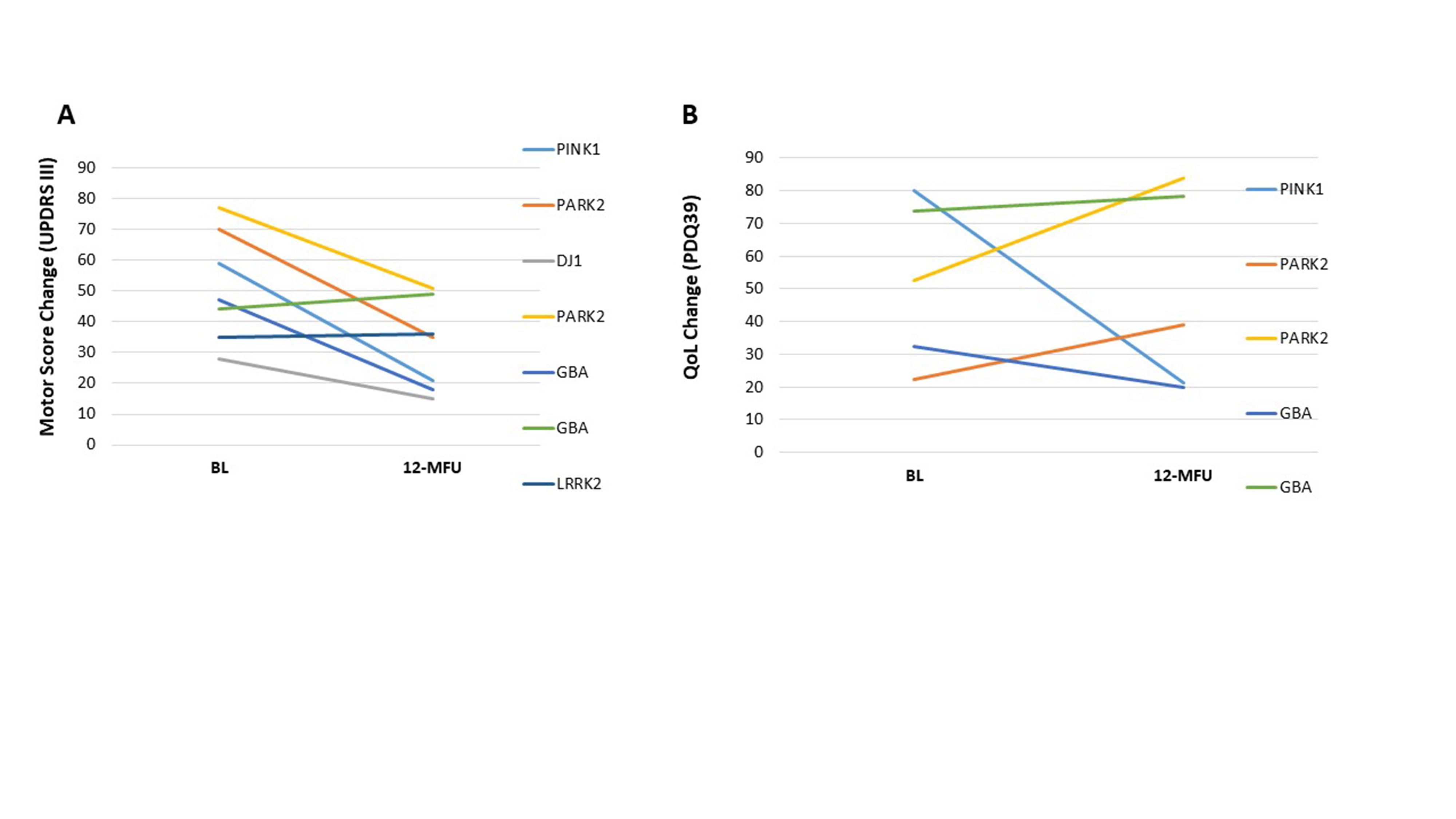

Supplement: Supplementary file 1 [file Image_1.JPEG]
